# Intracholecystic versus Intravenous Indocyanine Green (ICG) Injection for Biliary Anatomy Evaluation by Fluorescent Cholangiography during Laparoscopic Cholecystectomy: A Case–Control Study

**DOI:** 10.3390/jcm11123508

**Published:** 2022-06-17

**Authors:** Lidia Castagneto-Gissey, Maria Francesca Russo, Alessandra Iodice, James Casella-Mariolo, Angelo Serao, Andrea Picchetto, Giancarlo D’Ambrosio, Irene Urciuoli, Alessandro De Luca, Bruno Salvati, Giovanni Casella

**Affiliations:** 1 Department of Surgical Sciences, Sapienza University of Rome, 00161 Rome, Italy; lidia.castagnetogissey@uniroma1.it (L.C.-G.); mfrancesca.russo@outlook.com (M.F.R.); alessandraiodice@live.com (A.I.); irene.urciuoli@gmail.com (I.U.); dr.aless.deluca@gmail.com (A.D.L.); bruno.salvati@uniroma1.it (B.S.); 2 Department of General and Emergency Surgery, Ospedale dei Castelli (NOC), ASL Roma 6, 00040 Rome, Italy; jamescasella@hotmail.it (J.C.-M.); angelo.serao@aslroma6.it (A.S.); 3 Department of General Surgery, Surgical Specialties and Organ Transplantation, Sapienza University of Rome, 00161 Rome, Italy; andrea.picchetto@uniroma1.it (A.P.); giancarlo.dambrosio@uniroma1.it (G.D.)

**Keywords:** laparoscopic cholecystectomy, indocyanine green, ICG, intraoperative cholangiography

## Abstract

(1) Background: Fluorescence cholangiography has been proposed as a method for improving the visualization and identification of extrahepatic biliary anatomy in order to possibly reduce injuries and related complications. The most common method of indocyanine green (ICG) administration is the intravenous route, whereas evidence on direct ICG injection into the gallbladder is still quite limited. We aimed to compare the two different methods of ICG administration in terms of the visualization of extrahepatic biliary anatomy during laparoscopic cholecystectomy (LC), analyzing differences in the time of visualization, as well as the efficacy, advantages, and disadvantages of both modalities. (2) Methods: A total of 35 consecutive adult patients affected by acute or chronic gallbladder disease were enrolled in this prospective case–control study. Seventeen patients underwent LC with direct gallbladder ICG injection (IC-ICG) and eighteen subjects received intravenous ICG administration (IV-ICG). (3) Results: The groups were comparable with regard to their demographic and perioperative characteristics. The IV-ICG group had a significantly shorter overall operative time compared to the IC-ICG group (*p* = 0.017). IV-ICG was better at delineating the duodenum and the common hepatic duct compared to the IC-ICG method (*p* = 0.009 and *p* = 0.041, respectively). The cystic duct could be delineated pre-dissection in 76.5% and 66.7% of cases in the IC-ICG and IV-ICG group, respectively, and this increased to 88.2% and 83.3% after dissection. The common bile duct could be highlighted in 76.5% and 77.8% of cases in the IC-ICG and IV-ICG group, respectively. Liver fluorescence was present in one case in the IC-ICG group and in all cases after IV-ICG administration (5.8% versus 100%; *p* < 0.0001). (4) Conclusions: The present study demonstrates how ICG-fluorescence cholangiography can be helpful in identifying the extrahepatic biliary anatomy during dissection of Calot’s triangle in both administration methods. In comparison with intravenous ICG injection, the intracholecystic ICG route could provide a better signal-to-background ratio by avoiding hepatic fluorescence, thus increasing the bile duct-to-liver contrast.

## 1. Introduction

Cholecystectomy represents one of the most commonly performed laparoscopic operations on a global scale [1]. Despite the fact that laparoscopic cholecystectomy is now the gold standard for benign gallbladder surgery, it is still associated with a non-negligible incidence of bile duct injury, often owing to a misinterpretation of biliary anatomy [2,3]. Furthermore, while identifying the common bile duct (CBD) is normally straightforward in elective cholecystectomies, it can become difficult and challenging when severe inflammation and adhesions at the gallbladder hilum cause the distortion of the anatomy, making the identification of the CBD extremely demanding.

As a result, several techniques have been proposed to clearly envision and examine the extrahepatic biliary anatomy during surgery, such as employing routine intraoperative cholangiography. This procedure has reportedly been shown to decrease the rate of bile duct injury; however, it is seldom implemented due to its costs, augmented operative time, and need for additional specific instrumentation [3,4]. In the quest to reduce this type of complication, Strasberg et al. proposed the ‘critical view of safety’ approach, although its effectiveness in reducing bile duct injuries has not been unanimously confirmed [5].

The use of a fluorescence imaging system through the administration of indocyanine green (ICG) has been proposed as a method for improving the visualization and identification of extrahepatic biliary anatomy in order to possibly reduce injuries and related complications. The most common method of ICG administration is the intravenous route, as this drug is uniquely excreted through bile, appearing fluorescent when visualized through a near-infrared camera system. Several authors have described the usefulness of performing intraoperative fluorescence cholangiography with intravenous ICG injection, demonstrating how it facilitates the surgeon’s task of correctly highlighting and recognizing vital biliary structures even before any dissection takes place [6,7,8,9]. The direct injection of ICG into the gallbladder has also been proposed to achieve fluorescence cholangiography, although evidence on this modality is still quite limited [10,11,12,13,14,15,16].

We aimed to compare the two different methods of ICG administration—namely, the intravenous or intracholecystic route—for a more precise visualization and delineation of extrahepatic biliary anatomy during laparoscopic cholecystectomy, analyzing differences in the time of visualization, as well as the efficacy, advantages, and disadvantages of both modalities.

## 2. Materials and Methods

### 2.1. Study Design

This prospective study was carried out between September 2021 and March 2022 at the Department of Surgical Sciences, Sapienza University of Rome. It included 35 consecutive adult patients affected by acute or chronic gallbladder disease. Patients were subdivided into two groups: 18 patients in the laparoscopic cholecystectomy with intravenous ICG administration (IV-ICG) group and 17 patients in the intracholecystic ICG administration (IC-ICG) group. Patients meeting the inclusion criteria were consecutively recruited for the study, matched, and then randomly allocated into either group.

The inclusion criteria comprised age >18 years and the presence of symptomatic gallstone disease or acute/chronic cholecystitis. The exclusion criteria included known allergy to iodides, any previous major abdominal surgery, liver disease/failure, pregnancy, or breastfeeding. Patients with clinical suspicion of gallbladder malignancy were excluded from the study due to the risk of bile spillage, especially in the IC-ICG group with the consequent possibility of peritoneal seeding of cancer cells.

Institutional review board and ethical committee approval was obtained. All participants provided written informed consent to participate in the study. Additional written informed consent was obtained for all surgical procedures and for ICG administration.

### 2.2. Outcomes

The primary outcome was to evaluate the best method of delineating biliary anatomy amongst the two groups. The secondary outcomes included operative time, perioperative complications, and adverse events related to the ICG administration mode.

### 2.3. Intracholecystic ICG Administration

**Figure 1:**
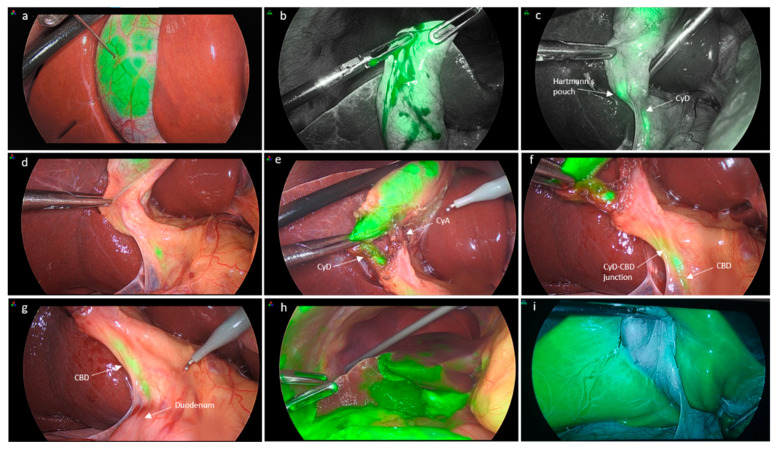
Intracholecystic ICG injection. (**a**) ICG injection by direct puncture of the gallbladder with a 27-gauge needle; (**b**) minimal spillage of ICG at the level of the puncture site, which is readily grasped to prevent further spillage; (**c**) milking of the gallbladder to allow for delineation of distal bile ducts; (**d**) ICG visualization prior to dissection and (**e**,**f**) after dissection of Calot’s triangle; (**g**) the common bile duct is clearly visualized also in its distal portion; (**h**) view of the abdominal cavity after a case of significant ICG spillage; and (**i**) erroneous administration of ICG in the gallbladder wall rather than in its lumen causing one case of hepatic fluorescence in the IC-ICG (CyD: cystic duct, CyA: cystic artery, CBD: common bile duct).

ICG (Verdye 25 mg/5 mL) was diluted in 10 mL of distilled sterile water. The gallbladder was grasped at the level of its fundus and retracted cephalad, bringing it to the proximity of the anterior abdominal wall. At this point, a 27-gauge needle was used percutaneously to puncture the abdominal wall and the fundus of the gallbladder in order to inject ICG as necessary to reach appropriate fluorescence levels (Figure 1a). The amount injected was variable depending on the dimensions of the gallbladder and the density of the bile. An average 5 mL of diluted ICG was usually necessary in our group of patients. Once the needle was removed, the introduction whole was readily grasped so as to reduce the risk of ICG spillage, which could hamper the clear visualization of fluorescence within the biliary structures.

In the IC-ICG group, in the presence of hydrops, the gallbladder content was aspirated and was then injected with ICG, whereas if a gallstone was impacted at the level of the infundibulum, the stone was tentatively mobilized upwards in order to allow ICG to flow downstream towards the common bile duct. The milking of the stone cephalad was carried out repeatedly so as to allow ICG to flow through the cystic duct and into the CBD.

### 2.4. Intravenous ICG Administration

**Figure 2:**
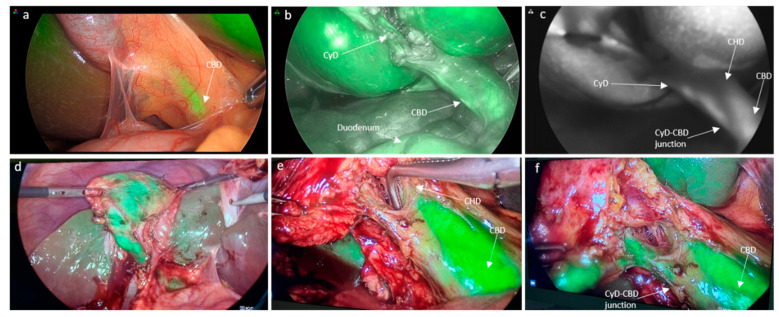
Intravenous ICG injection. (**a**) Initial view of ICG-cholangiography prior to dissection; (**b**) visualization of cystic duct after dissection; (**c**) view in ‘SPY’ mode; (**d**) clear delineation of the gallbladder; and (**e**,**f**) cystic and common bile duct with minimal liver fluorescence (CyD: cystic duct, CyA: cystic artery, CBD: common bile duct, CHD: common hepatic duct).

As ICG is entirely cleared by the liver and achieves a good concentration in the bile when given well in advance, ICG was administered intravenously approximately 45 min prior to surgery at a concentration of 0.01 mg/kg (Figure 2).

### 2.5. Surgical Procedure

Patients were positioned supine with split legs in the so-called ‘French position’. Four trocars were used: one 12 mm umbilical port and three 5 mm ports below the right subcostal margin. After ICG administration via the intravenous or intracholecystic route, the gallbladder was retracted cranially and the hilum was exposed. Fluorescence with an overlay or gray scale mode was used to assess biliary anatomy before any dissection was performed. A Pinpoint Fluorescence Endoscopic Imaging System (Novadaq/Stryker, Burnaby, BC, Canada) was used for all procedures. The dissection began on the lateral aspect of the infundibulum and was then continued medially. A critical view of safety approach was used in all cases. Once all elements of Calot’s triangle were visualized, the imaging quality of fluorescent biliary structures was reassessed once more and recorded. The cystic duct and artery were ligated with titanium clips and a retrograde cholecystectomy was completed.

Two individual surgeons independently evaluated the appearance of the ICG fluorescence cholangiography. The results were recorded and analyzed.

Postoperative pain was evaluated by means of a visual analogue scale (VAS) as recorded routinely in our clinical practice. Pain was divided into mild (1–3), moderate (4–6), and severe (7–10) categories.

### 2.6. Statistical Analysis

Continuous variables are expressed as mean ± SD in the case of normal distribution or medians. Normality was assessed using histograms and the Shapiro–Wilk test. Ordinal (semi-quantitative scores) and non-ordinal categorical variables are expressed as numbers (percentage).

All changes were compared by means of a *t*-test or a Mann–Whitney U test.

The association between IC-ICG and IV-ICG and the ability to delineate the bile duct anatomy was tested by means of a chi-squared test or a Fisher exact test if appropriate.

SPSS version 27 was used (IBM Corp. Released 2020. IBM SPSS Statistics for Windows, Version 27.0. IBM Corp, Armonk, NY, USA).

## 3. Results

### 3.1. Characteristics of the Study Population

Thirty-five patients undergoing laparoscopic cholecystectomy for benign gallbladder disorders were included in the analysis. The study group comprised 17 cases of laparoscopic cholecystectomy with ICG administered with direct gallbladder injection (IC-ICG) and 18 cases of laparoscopic cholecystectomy with ICG administered intravenously (IV-ICG). Both groups were comparable with regard to their demographic and perioperative characteristics (Table 1).

Cholecystitis was the indication for surgery in 14 patients (82.3%%) in the IC-ICG group and in 15 patients (83.4%) in the IV-ICG group. In total, 1 patient (5.9%) in the IC-ICG group had adenomyomatosis of the gallbladder, whereas 2 patients (11.8%) had choledocholithiasis and required concomitant ERCP during surgery.

The two groups were comparable with regard to their demographics and comorbid conditions, except for GERD, which had a higher incidence in the IC-ICG group. Additionally, hydrops was more common in the IC-ICG group compared to the IV-ICG group (41.2% versus 5–6%, respectively; *p* = 0.003).

### 3.2. Ability to Delineate Bile Duct Anatomy

Table 2 summarizes the ability of ICG to delineate the bile duct anatomy according to the administration method. IV-ICG was better at delineating the duodenum and the common hepatic duct (CHD) (22.2% versus 5.9%) compared to the IC-ICG method (*p* = 0.009 and *p* = 0.041, respectively). On the other hand, the ability to visualize the gallbladder, cystic duct (CyD) pre- and post-dissection, the common bile duct (CBD), and the CyD-CBD confluence were similar between the two groups. In particular, the gallbladder could be visualized by ICG-fluorescence in 88.2% and 88.9% of cases in the IC-ICG and IV-ICG group, respectively. There were two cases in which the gallbladder could not be assessed by fluorescence in the IC-ICG group due to technical errors, one being consequent to the ICG injection within the gallbladder wall (Figure 1i) and the other being caused by a puncture of the gallbladder wall from part to part, with ICG spillage in the peritoneal cavity. The cystic duct could be delineated pre-dissection in 76.5% and 66.7% of cases in the IC-ICG and IV-ICG group, respectively, and this increased to 88.2% and 83.3% after dissection. The common bile duct (CBD) could be highlighted in 76.5% and 77.8% of cases in the IC-ICG and IV-ICG group, respectively. Liver fluorescence was present in one case in the IC-ICG group and in all cases in the IV-ICG administration group (5.8% versus 100%; *p* < 0.0001). In the IC-ICG group there were two (11.8%) cases in which none of the biliary structures were visualized, coinciding with the two patients with hydrops.

In the IC-ICG group, the timing of visualization of biliary structures after direct gallbladder injection of ICG was an average 54.3 ± 38.5 s to delineate the CyD, 101.3 ± 50.6 s for the CyD-CBD junction, 164.2 ± 53.1 s for the CBD, and 272.3 ± 21.07 s for the duodenum (Table 3).

### 3.3. Perioperative Outcomes

No adverse event or bile duct injury was recorded in either group. No intraoperative complications occurred, whereas one (5.6%) patient developed acute pancreatitis 15 days postoperatively in the IV-ICG group.

The IV-ICG group had a significantly shorter overall operative time compared to the IC-ICG group (*p* = 0.017) (Table 4). Bile/ICG spillage was substantially greater in the IC-ICG group (64.7% versus 5.6%, respectively; *p* = 0.001). Spillage was a consequence of leakage through the injection site at the level of the gallbladder fundus in the IC-ICG group or due to the perforation of the gallbladder during dissection in either group. Spillage hampered the visualization of biliary ducts in only one patient in which spillage of bile/ICG was significant (Figure 1h). The spillage of gallbladder content was associated with postoperative pain in 23.5% of patients in the IC-ICG group versus none in the IV-ICG group (*p* = 0.001).

## 4. Discussion

Intraoperative fluorescence cholangiography can represent a rapid, non-invasive, technically simple modality for achieving real-time cholangiographic images, allowing for a safer biliary dissection even in the presence of anatomical variants. Two routes of ICG administration have been described so far, although there are no studies to date actively comparing these two different approaches.

There is growing evidence regarding the use of ICG fluorescence cholangiography via the intravenous pathway [9]. Systemic administration leads to the concentration of ICG within hepatocytes and its subsequent excretion through bile. The passage of ICG through the liver first, however, is responsible for the reduction in the signal-to-background ratio, translating into a high interference of the liver brightness with the visualization of extrahepatic biliary structures [10]. Furthermore, biliary excretion, especially in patients with liver dysfunction, is not predictable and can influence the fluorescence output. Although this interference is undeniable, several strategies could contribute to the optimization of bile duct delineation. The timing of preoperative intravenous injection together with an appropriate ICG dosage is crucial to obtain adequate imaging. Zarrinpar et al. tested different doses of intravenous ICG and concluded that the optimal dosage is 0.25 mg/kg 45 min preoperatively [11]. In the present study, we used an even lower dose (0.01 mg/kg) with the same timing, which allowed us to reach an excellent visualization of extrahepatic bile ducts with little to no interference with liver brightness. On the contrary, Verbeek et al., by trying different ICG concentrations (5, 10, 20 mg) and administration timings (30 min versus 24 h preoperatively), found that an intermediate dose (10 mg) with delayed timing to surgery (24 h) was the ideal combination to achieve negligible hepatic background fluorescence [12]. The timing and dosage issues still need to be properly assessed through well-designed studies in order to standardize intravenous ICG cholangiography.

The direct injection of ICG into the gallbladder could improve the visualization of the biliary tree by avoiding its hepatic flow, hence the background liver fluorescence. Graves et al. were the first to describe the direct gallbladder injection of ICG in 11 patients. However, the study was performed in a small, single-arm group of patients without any control group to compare outcomes with [13]. The authors concluded that this method was useful for the immediate delineation of extrahepatic biliary structures, also clarifying the plane of dissection between the gallbladder and hepatic bed.

Similarly, Liu et al. used the same route of ICG injection in a larger series of subjects undergoing laparoscopic cholecystectomy [10]. This was performed in two different ways, either through a previously placed percutaneous transhepatic gallbladder catheter in 18 patients or by intraoperative percutaneous gallbladder needle puncture in 28 subjects. The authors concluded that cholecystocholangiography was beneficial compared to white light alone in recognizing bile ducts, especially in the presence of gallbladder inflammation.

Skrabec et al. compared laparoscopic cholecystectomy with IC-ICG administration to the standard approach with no ICG-cholangiography, finding no significant differences between groups in terms of operative time and perioperative complications while underlining the usefulness of IC-ICG for outlining the biliary tree [14].

In a prospective single-arm study by Cardenas et al., 23 patients underwent direct gallbladder ICG injection and were able to demonstrate an optimal critical view of safety in all cases [15].

Shibata et al. described the differences between intravenous ICG administration versus intrabiliary administration with 12 patients in each group [16]. However, the ICG administration in the intrabiliary group was performed in three different ways: via transhepatic gallbladder drainage in 8 patients, through an endoscopic nasobiliary drainage in 1 patient, and by direct gallbladder injection in 3 patients. The non-standardized intrabiliary approach does not allow us to draw definitive conclusions on the superiority of one modality over another.

The only randomized controlled trial published in the literature was reported by Dip et al., who compared the efficacy of near-infrared fluorescent cholangiography to white light alone in visualizing bile ducts and found that fluorescence was significantly superior in highlighting biliary structures pre-dissection compared to the latter group [17].

To the best of our knowledge, the present study represents the first report actively comparing and analyzing the differences between two methods of ICG administration with the aim of assessing the optimal modality for bile duct visualization, highlighting the advantages and disadvantages of each.

Both approaches demonstrated how fluorescence cholangiography can represent a helpful method for delineating the extrahepatic biliary anatomy, simplifying the identification of Calot’s triangle elements.

Compared to the IV-ICG administration mode, direct IC-ICG injection can provide a clearer visualization of the gallbladder and bile ducts, avoiding hepatic fluorescence and thereby enhancing the bile duct-to-liver contrast. In fact, liver fluorescence was significantly lower in the IC-ICG group compared to the IV-ICG group (5.8% versus 100%; *p* < 0.0001). The only case of hepatic fluorescence that occurred in the IC-ICG group was due to the erroneous administration of ICG in the gallbladder wall rather than in its lumen.

The IC-ICG method provided an intraoperative real-time cholangiography without the need to administer ICG prior to surgery. After ICG injection into the gallbladder, the CyD was visualized after only 54.3 ± 38.5 s, whereas it took 101.3 ± 50.6 s to delineate the CyD-CBD junction and 164.2 ± 53.1 s to see the CBD. However, the IV-ICG group had a significantly shorter overall operative time compared to the IC-ICG group (*p* = 0.017) due to the lack of need to surgically inject the ICG.

Another limitation of the IC-ICG modality was the case of hydrops, which appeared to represent a contraindication to direct gallbladder injection by not allowing the visualization of any of the biliary structures due to the impossibility of ICG flowing downstream towards the CBD. Additionally, bile/ICG spillage was substantially greater in the IC-ICG group (64.7% versus 11.1%, respectively; *p* = 0.001). Spillage was usually a consequence of leakage through the injection site at the level of the gallbladder fundus in the IC-ICG group or due to the perforation of the gallbladder during dissection in either group. The spillage of the gallbladder content was associated with postoperative pain in 23.5% of patients in the IC-ICG group versus none in the IV-ICG group (*p* = 0.001). This could probably be due to the greater concentration of ICG in bile after a direct intracholecystic injection compared to an intravenous one. In most cases, spillage was minimal and reduced the ability to recognize biliary structures in only one case.

IV-ICG was better at delineating the duodenum and the CHD compared to the IC-ICG method (*p* = 0.009 and *p* = 0.041, respectively). On the other hand, the ability to visualize the gallbladder, CyD pre- and post-dissection, the CBD, and the CyD-CBD confluence were similar between the two groups. In particular, the cystic duct could be delineated pre-dissection in 76.5% and 66.7% of cases in the IC-ICG and IV-ICG group, respectively, and this increased to 88.2% and 83.3% after dissection, whereas the CBD could be highlighted in 76.5% and 77.8% of cases in the IC-ICG and IV-ICG group, respectively.

## 5. Conclusions

The present study demonstrates how ICG-fluorescence cholangiography can be helpful in identifying the extrahepatic biliary anatomy during the dissection of Calot’s triangle after both administration methods. In comparison with intravenous ICG administration, the intracholecystic ICG route could provide a better signal-to-background ratio by avoiding hepatic fluorescence, thus increasing the bile duct-to-liver contrast. On the other hand, the intravenous route reduces operative time by avoiding surgical intraoperative administration and favors a better visualization of the proximal portion of the extrahepatic biliary tree. By improving biliary tree visualization, these approaches could contribute to lowering the risk of bile duct injury during laparoscopic cholecystectomy. Large randomized controlled trials are warranted in order to further confirm such outcomes.

## Figures and Tables

**Table 1:** Demographic and preoperative features of the study population.

	IC-ICG (n = 17)	IV-ICG (n = 18)	*p* Value
Sex, males	6 (35.3)	7 (38.9)	
Age, years	50 ± 19	55 ± 18	0.632
Weight, kg	67 ± 10	72 ± 14	0.297
BMI, kg/m^2^	24 ± 3	25 ± 3	0.248
Hypertension, n (%)	4 (23.5)	6 (33.3)	0.527
Diabetes, n (%)	1 (5.9)	0	0.303
CAD, n (%)	1 (5.9)	1 (5.6)	0.967
GERD, n (%)	4 (23.5)	0	**0.031**
Anticoagulants-antiaggregant, n (%)	2 (11.8)	1 (5.6)	0.518
Hydrops, n (%)	2 (11.8)	1 (5.6)	0.521
Sludge, n (%)	7 (41.2)	7 (38.9)	0.892
Stone dimensions, (mm)	2.75 ± 2.8	2.64 ± 3.4	0.925

(BMI: body mass index; CAD: coronary artery disease; GERD: gastroesophageal reflux disease).

**Table 2:** Ability of ICG to delineate bile duct anatomy according to the administration method.

	IC-ICG (n = 17)	IV-ICG (n = 18)	*p* Value
GB, n (%)	15 (88.2)	16 (88.9)	0.430
CyD pre-dissection, n (%)	13 (76.5)	12 (66.7)	0.612
CyD post-dissection, n (%)	15 (88.2)	15 (83.3)	0.298
CHD, n (%)	1 (5.9)	4 (22.2)	**0.041**
CyD-CBD confluence, n (%)	8 (47.1)	11 (61.1)	0.401
CBD, n (%)	13 (76.5)	14 (77.8)	0.935
Duodenum, n (%)	5 (29.4)	13 (72.2)	**0.009**
Liver fluorescence, n (%)	1 (5.9)	18 (100)	**0.001**

(GB: gallbladder; CyD: cystic duct; CBD: common bile duct; CHD: hepatic duct; CyD-CBD confluence: cystic duct-common bile duct confluence).

**Table 3:** Timing of visualization of biliary structures in the IC-ICG group.

	Visualization Timing (sec ± SD)
CyD identification	54.3 ± 38.5
CHD identification	215
CyD-CBD junction identification	101.3 ± 50.6
CBD identification	164.2 ± 53.1
Duodenum identification	272.3 ± 21.7

(CyD: cystic duct; CBD: common bile duct; CHD: common hepatic duct; CyD-CBD confluence: cystic duct-common bile duct confluence).

**Table 4:** Perioperative data.

	IC-ICG (n = 17)	IV-ICG (n = 18)	*p* Value
Duration of surgery (min)	87 ± 39	61 ± 13	**0.017**
Length of stay (days)	1 (0–5)	2 (1–3)	0.581
Spillage, n (%)	11 (64.7)	1 (5.6)	**0.001**
Intra-operative complications, n (%)	0 (0)	0 (0)	0.876
Postoperative complications, n (%)	0 (0)	1 (5.6)	0.240
Postoperative pain, n (%)	4 (23.5)	0 (0)	**0.001**

## Data Availability

Data will be shared by the corresponding author upon reasonable request.
